# Numerical study on the cerebral blood flow regulation in the circle of Willis with the vascular absence and internal carotid artery stenosis

**DOI:** 10.3389/fbioe.2024.1467257

**Published:** 2024-08-22

**Authors:** Hong Lv, Kailei Fu, Wei Liu, Zhiyi He, Zhiqing Li

**Affiliations:** ^1^ Department of Neurology, The First Affiliated Hospital of China Medical University, Shenyang, China; ^2^ Development and Related Diseases of Women and Children Key Laboratory of Sichuan Province, Chengdu, China; ^3^ Stroke Center, The First Affiliated Hospital of China Medical University, Shenyang, China; ^4^ School of Energy and Power Engineering, Dalian University of Technology, Dalian, China

**Keywords:** circle of Willis, vascular anatomical variations, flow characteristic, compensation function, hemodynamic factors

## Abstract

**Objectives:**

This study explores how vascular stenosis and absence affect the regulation of cerebral blood flow in the Circle of Willis (CoW) and the hemodynamic changes downstream of the stenosis.

**Materials and Methods:**

Forty idealized CoW models were simulated to analyze the impact of vascular absence and internal carotid artery (ICA) stenosis on hemodynamics. Inlet conditions were set using a physiological pressure waveform, and outflow boundaries were modeled using three-element Windkessel models.

**Results:**

The absence of vessels such as RP1, LP1, RA1, or LA1 had a comparable effect on total blood flow to a 40% stenosis of the left internal carotid artery (LICA) across the entire CoW. Specifically, when LP1 and RA1 were absent with a 50% LICA stenosis, the total blood flow closely resembled that of a complete CoW with 75% LICA stenosis. In cases of proximal ICA stenosis, downstream regions showed elevated oscillatory shear index (OSI >0.2) and reduced time-averaged wall shear stress (TAWSS <1 Pa). With increasing stenosis severity, areas of high OSI shifted, and regions of low TAWSS expanded notably. At 75% stenosis, the area with TAWSS <1 Pa downstream significantly increased. Until complete occlusion, the area of low TAWSS and high OSI were maximized.

**Conclusion:**

This study underscores how anatomical variations in the CoW, combined with ICA stenosis, impact both total cerebral blood flow and its distribution among different outlets. Moreover, it highlights the potential for increased atherosclerosis development in affected areas. Particularly notable is the finding the absence of LP1 and RA1 vessels alongside 50% LICA stenosis results in blood flow patterns similar to those seen with 75% LICA stenosis in a complete CoW, emphasizing clinical implications for the patient. Hemodynamic changes, including TAWSS and OSI, are most pronounced downstream of the stenosis especially when the stenosis rate exceeds 75%.

## 1 Introduction

The Circle of Willis (CoW) is a crucial network of cerebral arteries encircling the base of the brain, serving as the primary collateral circulation for the cerebral blood supply. Its integrity is pivotal for maintaining optimal brain function. A complete CoW typically comprises twelve main cerebral vessels, including two internal carotid arteries (ICAs), two anterior cerebral arteries (ACAs), two middle cerebral arteries (MCAs), the basilar artery (BA), two posterior cerebral arteries (PCAs), and three communicating arteries (Alpers et al., 1959).

Variations in CoW anatomy are common among the general population, with more than half of individuals having deviations from the complete structure (Kapoor et al., 2008; Li et al., 2011). These variations encompass approximately 23 different anatomical configurations, ranging from mild malformations to the complete absence of specific vessels (Eftekhar et al., 2006). Such variations, alongside arterial stenosis, significantly influence cerebral perfusion and are closely linked to cerebral ischemic events. For instance, severe ICA stenosis or occlusion places greater reliance on CoW collateral circulation, increasing the risk of cerebral infarction compared to individuals with a normal CoW (Alpers and Berry, 1963; Battacharji et al., 1967). In this complex interplay, the function of cerebral collateral circulation, particularly through the circle of Willis (CoW) was crucial (Desai et al., 2021; Neidlin et al., 2023).

Recent studies employing one-dimensional computer modeling have explored the impact of CoW anatomical variations and occlusions on cerebral blood flow dynamics. Researchers have investigated scenarios such as hyperperfusion following carotid artery surgery is related to CoW anatomy and severity of ICA stenosis (Liang et al., 2011; Liang et al., 2009). These studies highlight the complex interplay between anatomical variations, stenosis severity, and cerebral perfusion dynamics.

Advancements in medical imaging and computational fluid dynamics (CFD) have further enabled the study of patient-specific cerebral flow characteristics. Studies have evaluated hemodynamic parameters within CoW models under varying degrees of stenosis, revealing significant pressure discrepancies and altered flow patterns in response to arterial narrowing (Long et al., 2008; Sun et al., 2024). Such investigations underscore the critical role of CoW in cerebral blood regulation and highlight its adaptive capacity to compensate for vascular deficiencies.

Despite these advancements, there remains a gap in understanding how morphological variations within CoW affect global cerebral blood regulation, particularly based on *in vitro* experimentation. Existing studies predominantly focus on normal CoW function or specific anatomical configurations (Cieslicki and Ciesla, 2005; Cieślicki, 2004), limiting comprehensive insights into the broader implications of CoW variability on cerebral blood flow regulation.

Therefore, this study aims to fill this gap by numerically assessing the regulatory capacity of cerebral blood flow in CoW across eight anatomical variations combined with varying degrees of ICA stenosis using idealized CoW models. By quantitatively analyzing cerebral blood flow distributions under conditions of stenosis and vessel absence, this research seeks to enhance understanding of CoW’s blood flow regulation and collateral circulation dynamics. Ultimately, findings may provide valuable insights for guiding treatment strategies and surgical planning for patients with cerebral ischemia.

## 2 Materials and Methods

### 2.1 Image acquisition and CoW model reconstruction

**FIGURE 1:**
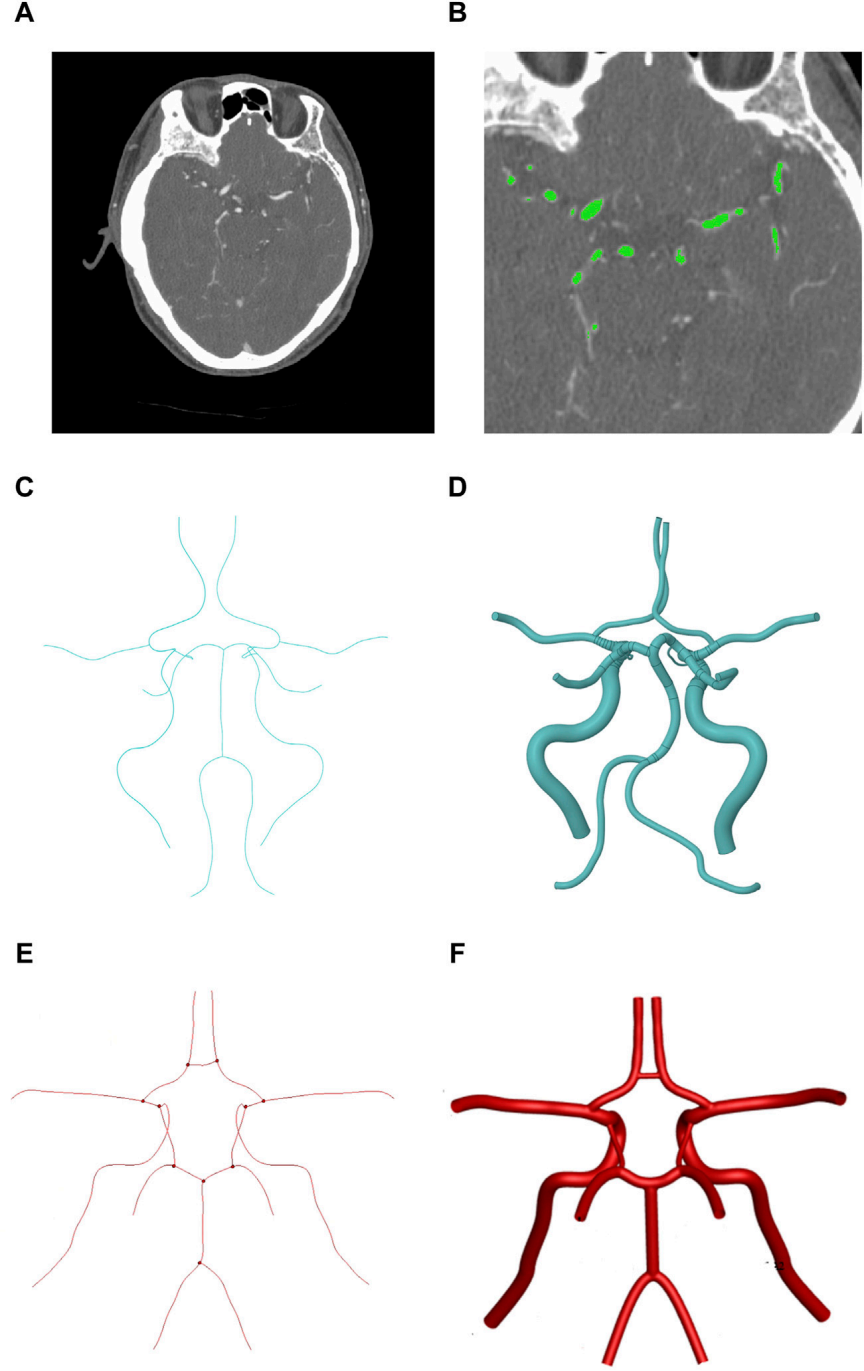
The progress of idealized geometric reconstruction of CoW. **(A)** Cross-sectional image with the brain. **(B)** Region detection of the CoW. **(C)** The patient-specific center line of CoW. **(D)** Patient-specific geometric reconstruction of CoW. **(E)** The symmetrical center line of CoW. **(F)** Symmetrical geometric reconstruction of CoW. (Medical imaging is a mirror process, so the right side in the image corresponds to the left side of the patient).

To obtain an idealized CoW model, the patient-specific CoW was first reconstructed. We used a set of CT angiography (CTA) scan data with the DICOM format (Figure 1A). All the original CT images came from a 20-year-old male volunteer, which were obtained from the First Affiliated Hospital of China Medical University. His CTA result showed no obvious vascular stenosis or cerebral vessel diseases. Most of the arteries appear whiter than the surrounding brain tissue. The CoW region (Figure 1B) is clearly shown in the CT images. Using the image processing module ScanIP of 3D reconstruction software (SIMPLEWARE Ltd., Exeter, UK), the region of CoW all images was successfully segmented based on grayscale intensity, and the subsequent 3D geometry was subsequently. For further refinement of the CoW model (Figure 1C), a recursive Gaussian filter was employed, which is a linear smoothing enhancement method. The CT image series had an inter-slice distance of 0.5 mm and an in-plane resolution of 0.64 mm. In this study, the patient-specific 3D CoW model involves 12 blood vessels: four inlets (2 VAs and 2 ICAs), three communicating arteries (2 PCOAs and 1 ACOA), and six outlets (2PCAs, 2MCAs and 2ACAs).

Then, the centerlines of each vessel were extracted automatically using the Vascular Modeling Toolkit (VMTK) (Cieslicki and Ciesla, 2005)which was exported in IGS format (Figure 1D), leaving the joint of vessels unconnected. We retained the annular vascular connection of CoW, simplified the spatial direction of the blood vessels of CoW, and symmetrized the CoW. An ideal symmetrical CoW model was obtained by using the average diameter of the blood vessels as its internal diameter. The internal diameters (ID) and lengths of all the branches of CoW in previous studies and our study are shown in Table 1.

**TABLE 1:** Internal diameters and lengths of all the branches of CoW in previous studies and our study.

	ID/length (mm)							
	ICA	BA	VA	ACA	MCA	PCA	ACoA	PCoA
Our study	4.0/98	3.2/20	2.8/25	2.5/35	3.5/20	3.0/30	1.5/4	1.5/13
Moore (Moore et al., 2006)	4.72/-	3.17/-	-	2.33/-	2.86/-	2.13/-	1.47/-	1.45/-
Yeniçeri I Ö (Yeniçeri et al., 2017)	4.24/-	2.85/-	-	1.60/-	2.13/-	1.8/-	-	1.12/-
Gunnal S A (Gunnal et al., 2014)	4.9/-	4.9/-	-	2.8/-	-	2.8/-	1.7/2.6	2.1/15.9

Figure 1 shows three cross-sectional images, the region segmentation, and its reconstructed 3D model acquired in CoW.

**FIGURE 2:**
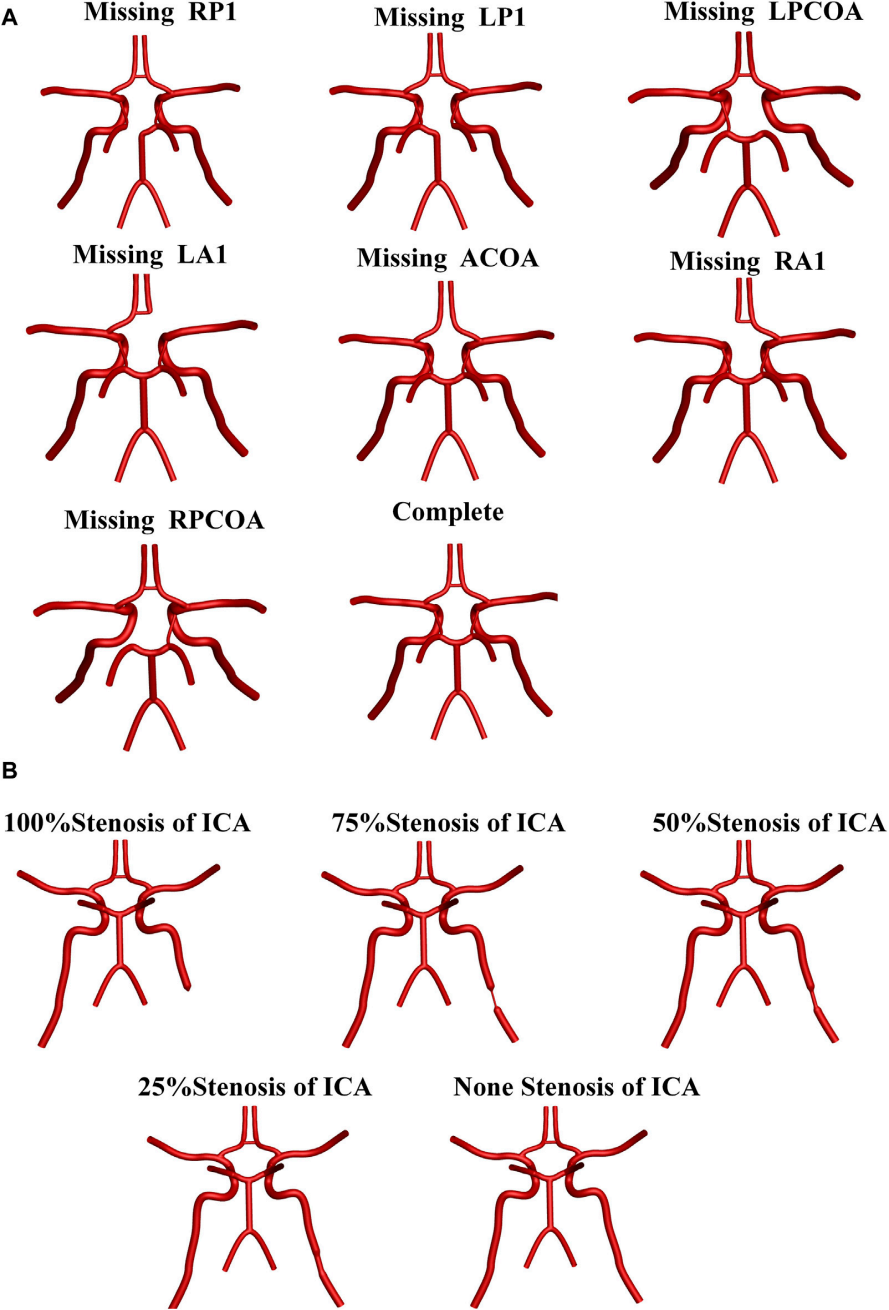
Main vascular anatomical variations of Cow in the idealized models. **(A)** Anatomical variations in the CoW branches. **(B)** Stenosis status of LICA.

To better analyze the compensation regulation of cerebral blood flow in CoW with the vascular anatomical variations, forty anatomical structural forms were applied in this study: a whole circle of Willis, and its seven vascular absence with five vessel stenosis (st: 0, 25%, 50%,75%, 100%) in the right internal carotid artery, respectively. The parameter of st was defined as follows: st = (1 − d/D) × 100%, where d and D are the diameters of the narrowest and normal regions, respectively (Cieslicki and Ciesla, 2005). Figure 2 shows the main vascular anatomical variation ns of CoW in the idealized models.

### 2.2 Boundary conditions and CFD simulations

ANSYS ICEM 2022R1 was used to generate unstructured meshes for the computational domain. Tetrahedral meshes were applied in the mainstream core region, with six layers of prismatic boundary layer meshes near the wall to capture boundary layer flows, where the first layer height of the boundary layer was set to 0.01 mm. Mesh independence tests were conducted on the complete CoW model without any artery stenosis. Three sets of grids with varying densities were selected for comparison, consisting of 730,000, 1,540,000, and 3,000,000 grid cells, respectively. By accounting for the second-order truncation error, the flow rates at the four outlets were statistically examined as metrics. It was observed that the relative variation in flow rate between the 3,000,000-cell grid and the 1,540,000-cell grid was below 0.5%. Hence, the study ultimately proceeded with the 1,540,000-cell grid for further investigations.

The three-dimensional flow inside blood vessels is computed using the Navier-Stokes equations based on momentum conservation and mass conservation principles. Since the Reynolds number of blood flow in cerebral vessels is below 2,300, blood is considered a Newtonian fluid exhibiting laminar flow characteristics. For the three-dimensional, incompressible flow of a Newtonian fluid, the following continuity (Equation 1) and Navier-Stokes (Equation 2) equations can be used to describe the flow:

∇⋅u=0 (1)

$$\left.\rho \left(\frac{\partial u}{\partial t}+u\cdot \nabla u\right)=-\nabla p+\mu \nabla^{2}u\right)$$ (2)

Where, μ represents the blood viscosity with a value of 3.5 mPa s, while ρ stands for density with a value of 1,060 kg/m³. Additionally, p denotes pressure, and u signifies the velocity vector of the fluid. Simulations are carried out using the commercial CFD solver ANSYS Fluent. The flow governing equations are solved using the finite volume method (FVM) (Caballero and Laín, 2013). A SIMPLE algorithm was adopted for coupling calculations of the pressure and velocity (Patankar and Spalding, 1983).

**FIGURE 3:**
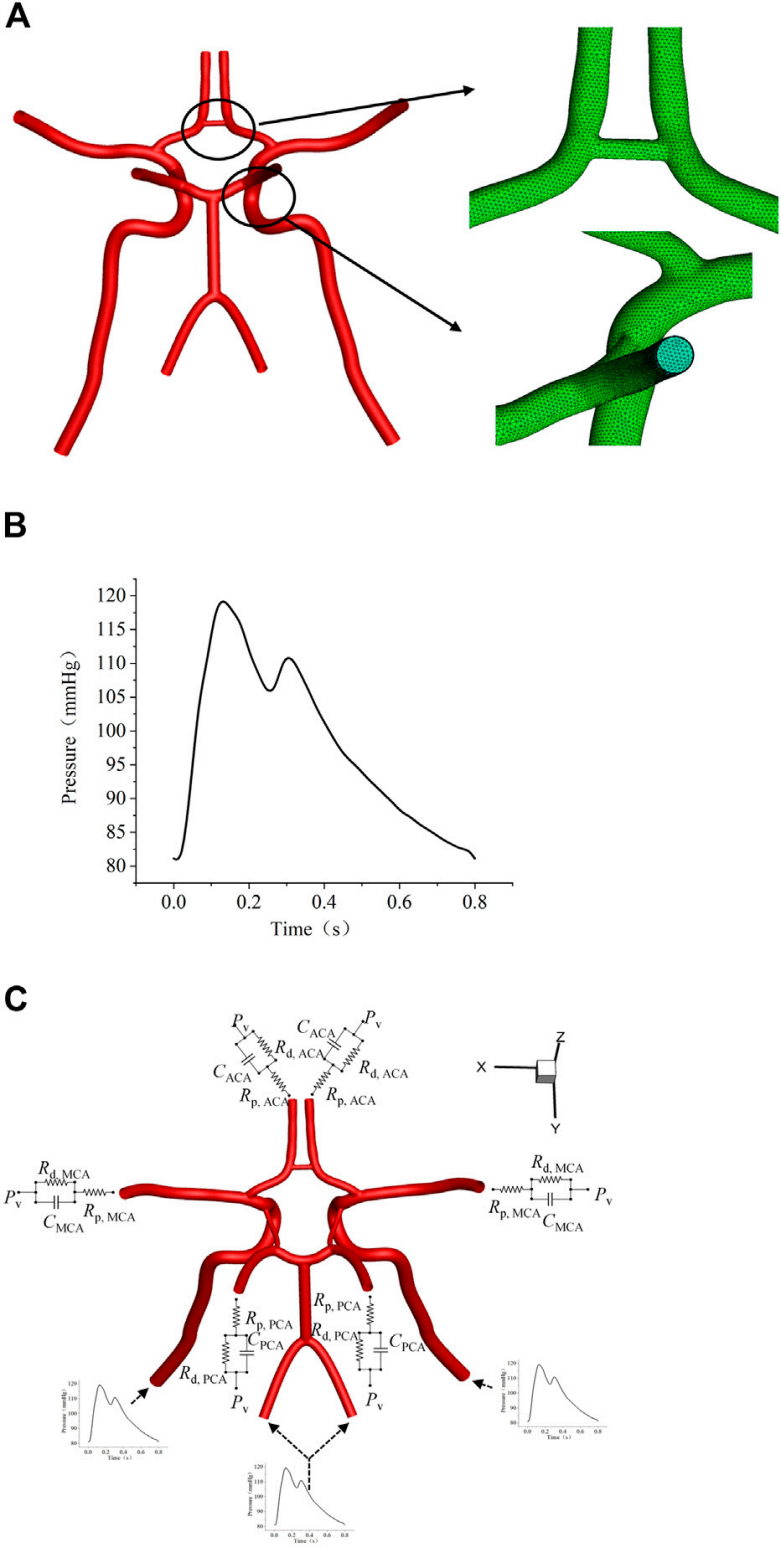
Settings of flow boundary conditions. **(A)** The layout of the polyhedral mesh model. **(B)** The waveform of the inlet boundary. **(C)** Schematic diagram of the 3-EWM.

Uniform boundary conditions settings were performed in all models of CoW. A pressure waveform reported by Zhu et al. (Zhu et al., 2015a), its cardiac cycle was 0.8 s, with a peak systole pressure of 120 mmHg (Figure 3B). A three-element Windkessel model (3-EWM, as illustrated in Figure 3C) was used for considering the influence of compliance in the computational domain and the distal vasculature, which consists of resistance Rp, compliance C, and distal resistance Rd. In this research, well-adjusted parameters of the 3-EWM presented by Liang et al. (Liang et al., 2011) (Table 2), with pulse pressure in the patient-specific branchial artery and morphology similar to those of our samples, were adopted.

**TABLE 2:** Values of the 3-EWM adopted from Liang et al. (Liang et al., 2011).

	R_p_ [10^8^ kg m^-4^ s^-1^]	C [10−10 kg-1 m^4^ s^2^]	R_d_ [10^8^ kg m^-4^ s^-1^]
MCA	5.12	6.99	20.49
PCA	10.33	3.46	41.33
ACA	10.43	3.43	41.73

### 2.3 Data extraction and analysis

Time-averaged wall shear stress (TAWSS) represents the magnitude of the shear stress on the blood vessel wall throughout the cardiac cycle, which can be expressed by the formula as Equation 3 (Chi et al., 2021).

$$TA\text{WSS}=\frac{1}{T}\int_{0}^{T}\left|wss\left(s,t\right)\right|dt$$ (3)

Where T is a cardiac cycle period, while WSS is the transient wall shear stress at the position s.

The indicator OSI can be used to measure the changes in WSS, which can be expressed by the formula as Equation 4 (Chi et al., 2021).

$$OSI=\frac{1}{2}\left[1-\left(\frac{\left|\int_{0}^{T}wss\left(s,t\right)dt\right|}{\int_{0}^{T}\left|wss\left(s,t\right)\right|dt}\right)\right]$$ (4)

During the transient simulations, a second-order implicit time-stepping scheme with a fixed time discretization of 0.001 s was employed. Convergence was determined after four cardiac cycles when the results exhibited periodic fluctuations. An additional cardiac cycle was calculated for the extraction of transient data. During data processing, wall shear stress (WSS) at three coordinates was extracted every four time steps for generating TAWSS and OSI contours. Each calculation cycle encompassed 1,000 time steps, yielding a total of 250 datasets. The rectangular rule was employed for numerical integration in computing TAWSS and OSI.

## 3 Results

In this section, we present detailed comparisons of total blood flow rates and flow characteristics within the Circle of Willis (CoW), focusing on the influence of vascular anatomical variations and stenosis.

### 3.1 Total blood flow rate affected by vascular anatomical variations

**FIGURE 4:**
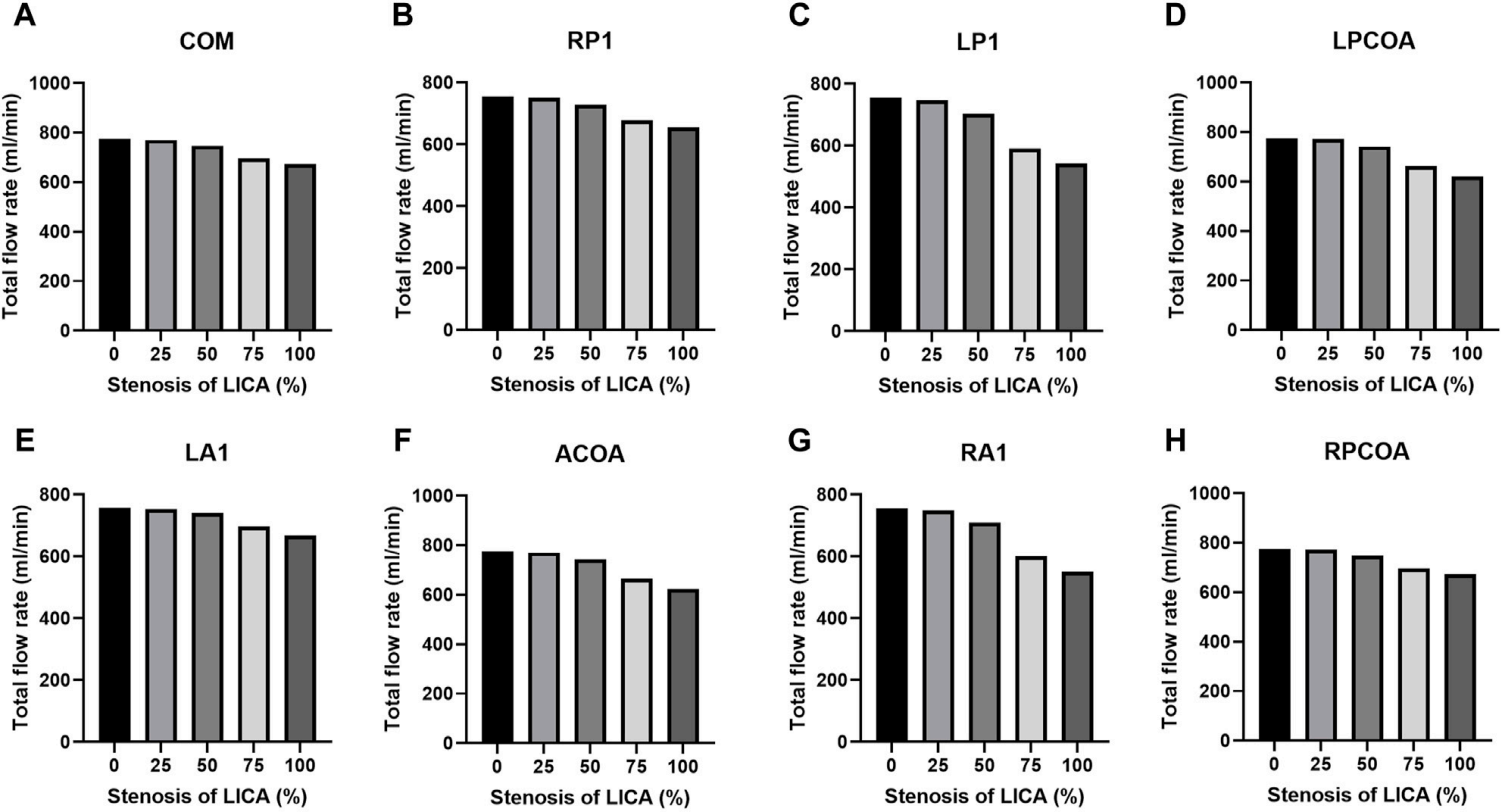
Total flow rates for the eight CoW configurations with increasing stenosis of LICA. **(A)** Complete COW. **(B)** RP1 missing. **(C)** LP1 missing. **(D)** LPCOA missing. **(E)** LA1 missing. **(F)** ACOA missing. **(G)** RA1 missing. **(H)** RPCOA missing.

**FIGURE 5:**
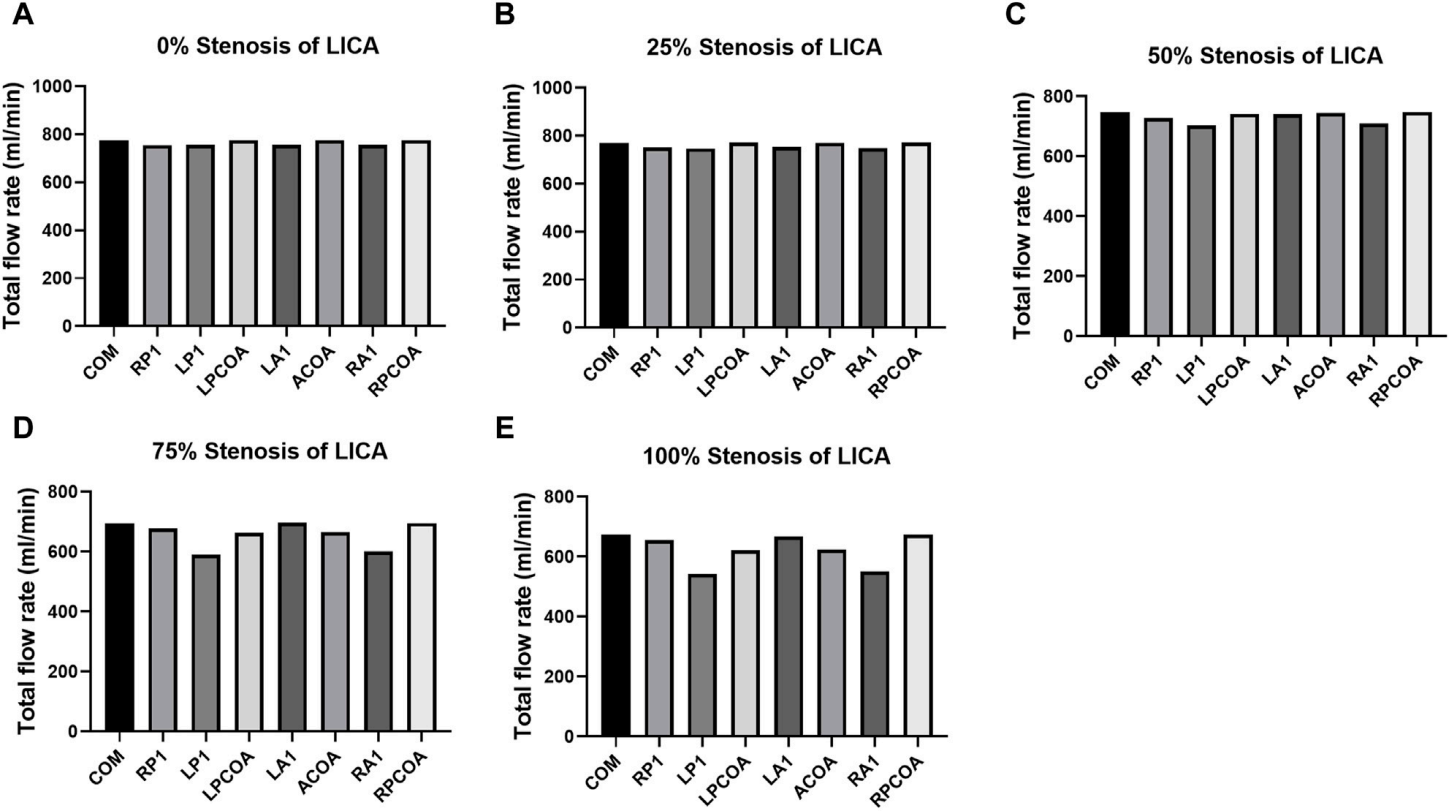
Total flow rates for the eight CoW configurations with increasing stenosis of LICA. **(A)** 0% Stenosis of LICA. **(B)** 25% Stenosis of LICA. **(C)** 50% Stenosis of LICA. **(D)** 75% Stenosis of LICA. **(E)** 100% Stenosis of LICA.

Table 3 lists the total flow rates for each CoW model studied, with the complete CoW without LICA stenosis serving as the reference. As expected, increasing degrees of blood vessel stenosis lead to increased downstream resistance and subsequent reductions in total flow rates, especially when the stenosis is above 50% (as shown in Figure 4). The absence of LP1 or RA1 alongside stenosis impacts the total CBF the most (as shown in Figure 5).

**TABLE 3:** Detailed total flow rates with different LICA stenosis degrees.

Stenosis degree, st (%)	Total flow rates (mL/min)							
	Complete	RP1 missing	LP1 missing	LPCOA missing	LA1 missing	ACOA missing	RA1 missing	RPCOA missing
0	775.23	754.90	755.15	775.61	756.47	775.50	755.90	775.70
25	770.47	750.02	746.77	771.07	753.96	770.29	749.02	771.12
50	747.13	727.92	703.71	741.67	740.29	744.01	708.80	747.71
75	695.15	677.36	590.43	662.72	696.07	665.58	600.88	695.92
100	673.30	654.69	541.86	621.53	667.70	623.44	550.18	674.05

**FIGURE 6:**
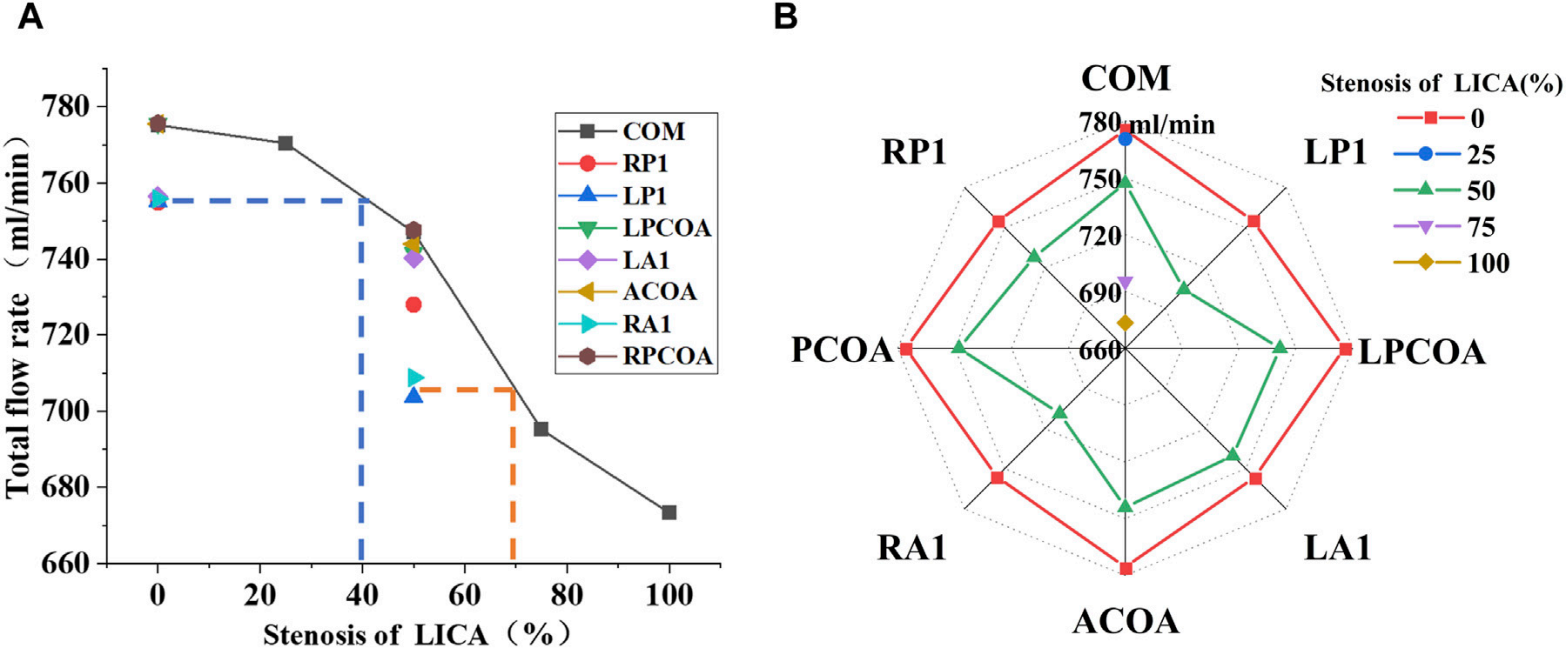
The coupling effect of vascular stenosis and absence on the total CBF. **(A)** Use a line chart to illustrate. **(B)** Use a radar chart to illustrate.

Figure 6 shows the coupling effect of vascular stenosis and absence on the total CBF. On the one hand, compared with the complete CoW without artery stenosis structure (as the broken line shows in the radar chart), the absence of blood vessels does not necessarily lead to a decrease in total blood flow. Such as in the case of the vascular missing of ACOA, LPCOA, and RPCOA, there are slight changes in the total CBF. However, when the absence of RP1, LP1, RA1, or LA1 occurs, it can reduce total CBF, which can be seen in the line chart. It seems to be that the effect of these four vascular variations on total CBF is equivalent to the one in the case of a 40% artery stenosis of LICA in the complete CoW. On the other hand, when LP1 and RA1 were absent with 50% LICA stenosis, the total blood flow closely resembled that of a complete CoW with 75% LICA stenosis.

**FIGURE 7:**
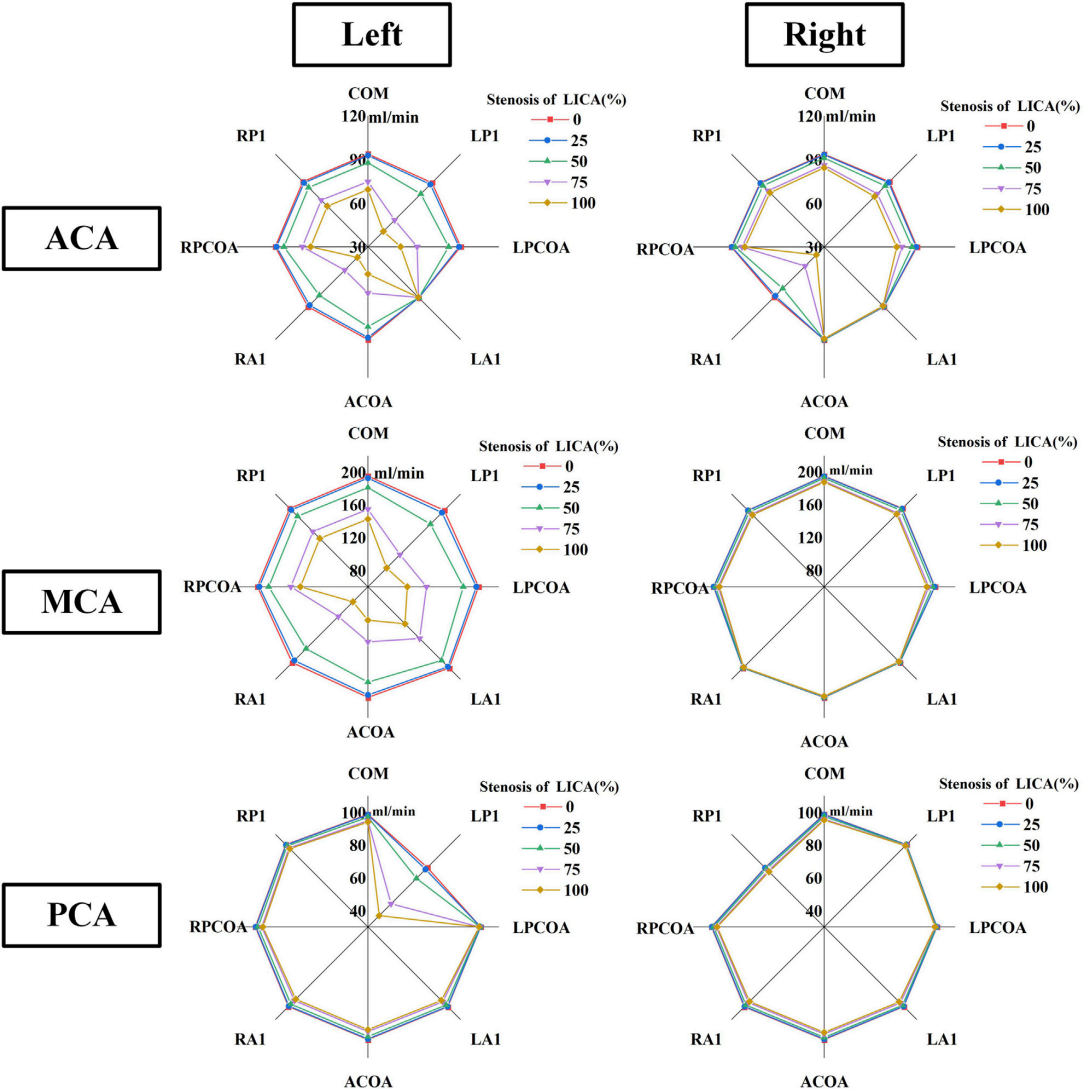
The blood rate tendency of six outlets in CoW on the ipsilateral and contralateral side of the artery stenosis.

Figure 7 shows the flow distribution tendency of the ACA outlet, PCA outlet, and MCA outlet on the ipsilateral and contralateral side of the artery stenosis. In models without RA1 or LP1, the reduction in blood flow rate at the left anterior cerebral artery (ACA) outlet was more pronounced with increasing LICA stenosis. Conversely, the absence of LA1 showed minimal impact on left ACA outflow despite varying degrees of LICA stenosis. Notably, the absence of RA1 significantly decreased blood flow at the right ACA outlet as both ACAs rely on blood supply from the LICA and basilar artery when RA1 is absent.

The absence of LP1 showed a more significant reduction in blood flow rate at the left posterior cerebral artery (PCA) compared to other variations. Conversely, the absence of the left posterior communicating artery (LPCoA) did not affect blood flow in the left PCA. With LP1 and LPCoA absent, the flow rate at the right PCA outlet remained unaffected by increasing LICA stenosis. When RP1 was absent, the greatest decrease in blood flow rate among all CoW variations was observed at the right PCA outlet. The absence of LP1 and RA1 led to a notable reduction in blood flow rate from the left middle cerebral artery (MCA) outlet, while the right MCA outlet showed minimal sensitivity to artery absence and stenosis.

### 3.2 OSI and TAWSS distribution affected by vascular anatomical variations

**FIGURE 8:**
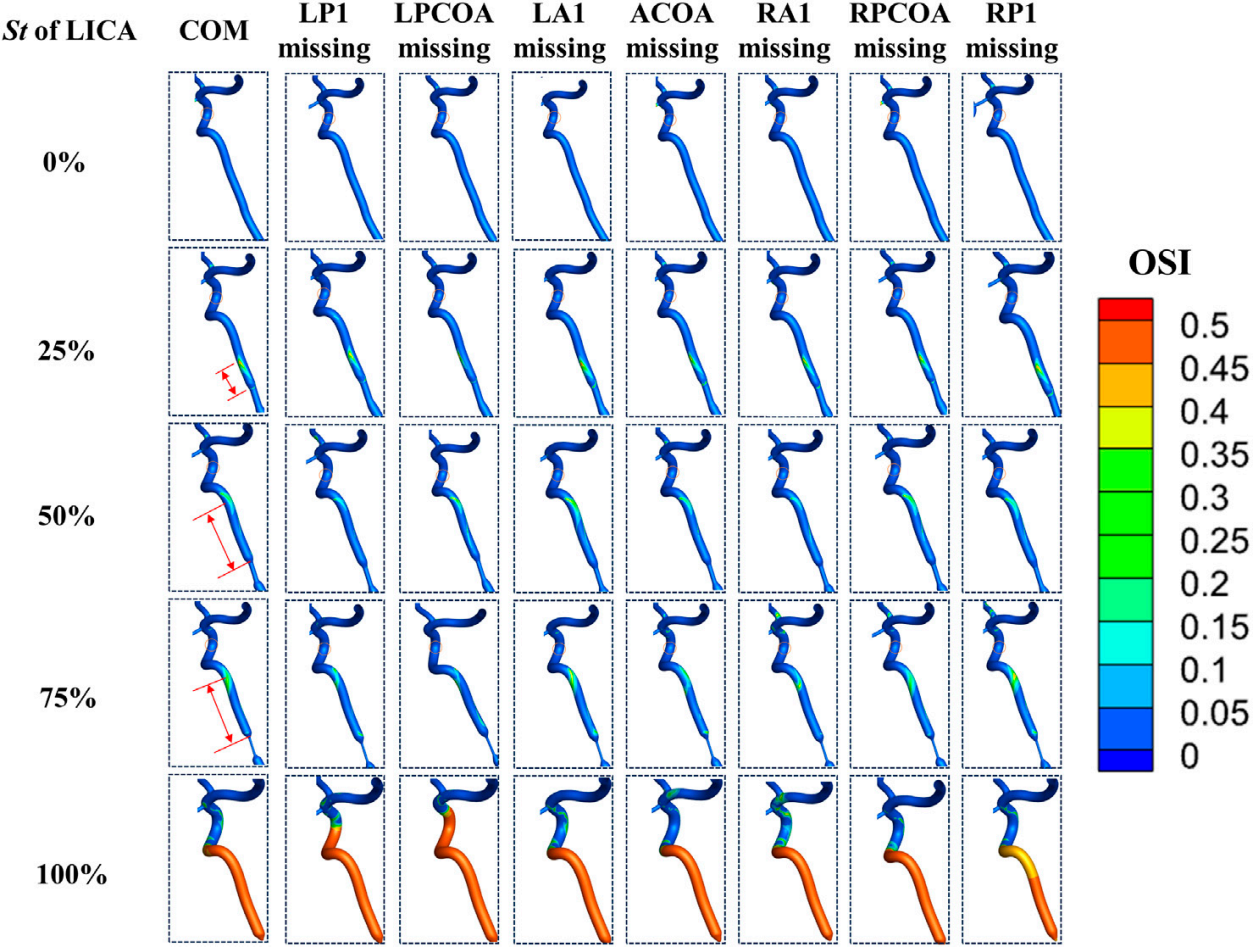
OSI distribution in the downstream vessels of the stenosis.

OSI values above 0.2 indicate regions where wall shear stress direction changes significantly, potentially predisposing to vascular pathology. Initially, without LICA stenosis, no prominent high OSI regions were observed downstream. However, with increasing stenosis severity, significant high OSI areas emerged downstream of the stenotic segment, shifting towards distal regions. This shift in OSI distribution signifies altered flow dynamics and potential endothelial stress. Notably, the bending part of the internal carotid artery (ICA) consistently exhibited high OSI regions across all cases, a known site prone to aneurysms or arterial plaque formation clinically (as shown in Figure 8).

**FIGURE 9:**
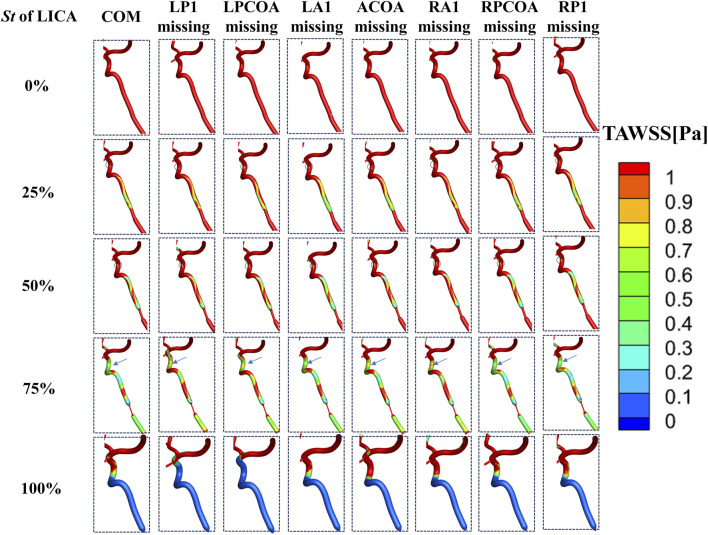
TAWSS distribution in the downstream vessels of the stenosis.

TAWSS, representing tangential stress on arterial walls by flowing blood, showed a consistent pattern of low TAWSS (<1 Pa) regions in the ICA’s bending part across stenosis levels of 25% and 50% (Figure 9). As LICA stenosis severity increased, the area of low TAWSS downstream expanded gradually. At stenosis levels exceeding 75%, a significant increase in low TAWSS areas was observed, indicating conditions where endovascular treatment might be warranted clinically. Low TAWSS (<1 Pa) has been linked to atherosclerotic plaque development (Davies and Civelek, 2011), suggesting its potential role in arterial sclerosis stenosis (as shown in Figure 9) (LaDisa et al., 2010).

## 4 Discussion

This study used the method of Computational Fluid Dynamics (CFD) to deeply explore the impact of vascular stenosis combined with vascular absence on cerebral blood flow, and quantitatively calculates total CBF and its distribution of different outlets. We used pressure distribution at the inlets in the CoW, including both ICAs and VAs. The outlets of the cerebral arteries were each connected to a three-element Windkessel model (RCR model) (Tian et al., 2024). The results are consistent with measured cerebral blood flow distribution by high-resolution nuclear magnetic resonance, indicating the feasibility of the calculation method (Zarrinkoob et al., 2015). The RCR calculation method adopted in our study has the advantage of exploring the hemodynamic changes of arterial stenosis combined with Willis variations under the same pressure boundary condition, which is the most consistent with the human physiological state. In previous studies, the velocity boundary condition was mostly used, but the entrance velocity was unknown when the vessel stenosis occurred, so it was not suitable for our study. The disadvantage of our calculated method is that it applies an idealized symmetric Willis model rather than an individualized model, which cannot fully simulate the diversity and heterogeneity of the human body.

This study discovered the absence of LP1 or RA1 combined with LICA stenosis decreased total CBF dramatically, suggesting LP1 and RA1 were of great significance in the collateral circulation regulation of CoW, which was partly reported previously (Zhu et al., 2015b). Interestingly, when LP1 or RA1 was absent alongside a 50% stenosis of LICA, the total blood flow mirrored that observed in a complete CoW with 75% LICA stenosis. This scenario underscores the clinical relevance, suggesting that such conditions may present similarly to cases of severe ICA stenosis in medical practice, and indeed, endovascular therapy or endarterectomy surgery might be involved in advance.

Our simulations revealed distinct patterns in OSI and TAWSS distributions across different CoW models with varying stenosis and vascular anatomical variations. With increasing stenosis severity, significant high OSI areas and low TAWSS emerged downstream of the stenotic segment, which are considered as characteristic hemodynamic parameters affecting vascular wall remodeling and inducing atherosclerosis development (Graham et al., 1992). We also found that at stenosis levels exceeding 75%, a significant increase in low TAWSS areas was observed, indicating conditions where endovascular treatment might be warranted clinically. However, in clinical practice, more attention is paid to the degree of vascular stenosis, in other words, the morphology of the blood vessels is emphasized. The results of this study remind us to simultaneously pay attention to the hemodynamic changes downstream of stenosis, as this may induce new arterial sclerosis stenosis, causing a further decrease of CBF. The impact of different anatomical variations on the areas of low TAWSS needs to be analyzed in detail.

## 5 Conclusion

In this study, we conducted numerical simulations to explore the hemodynamic implications of vascular stenosis and absence within an idealized Circle of Willis (CoW). Our findings shed light on the intricate dynamics of cerebral blood flow regulation under varying anatomical conditions.

The results highlight the significant impact of combined artery stenosis and absence on cerebral blood flow, particularly emphasizing the critical role of missing vessels like RA1 or LP1. These configurations were found to notably influence the overall blood flow dynamics in the CoW. Specifically, when LP1 and RA1 were absent alongside a 50% stenosis of the left internal carotid artery (LICA), the total blood flow mirrored that observed in a complete CoW with 75% LICA stenosis. This scenario underscores the clinical relevance, suggesting that such conditions may present similarly to cases of severe ICA stenosis in medical practice.

Moreover, our simulations revealed that the absence of RP1, LP1, RA1, or LA1 had a hemodynamic impact equivalent to a 40% stenosis of LICA across the entire CoW. This finding underscores the compensatory mechanisms within the CoW and highlights how anatomical variations can simulate physiological conditions of arterial stenosis.

We also observed distinct hemodynamic patterns downstream of proximal ICA stenosis, characterized by regions with elevated oscillatory shear index (OSI >0.2) and reduced time-averaged wall shear stress (TAWSS <1 Pa). As stenosis severity increased, these regions expanded significantly, particularly notable at 75% stenosis where the area of low TAWSS expanded markedly. Until complete occlusion, the area of low TAWSS and high OSI were maximized.

There are some limitations in our study. Firstly, the idealized model is adopted, but the morphology of the blood vessels in the human body varies a lot, and the conclusions obtained from the idealized model may not apply to specific individuals. Secondly, only five stenosis rates and eight vascular variants were calculated in our study, a total of 40 geometric models, and multiple stenosis or co-variations of CoW were not calculated, which is more common in the clinic. In addition, this study assumes that CoW is symmetrical, but in fact, the geometry of human cerebral blood vessels is variable and asymmetrical, and a large-sample numerical simulation study based on patient-specific geometry should be carried out in the future to obtain more meaningful conclusions. Furthermore, the inlet boundary conditions used in this study are pressure waveforms and the outlet boundary conditions are three-element Windkessel models, while the inlet and outlet boundary conditions, in reality, are difficult to be acquired accurately. Finally, in the numerical simulation calculation, it is considered that the blood vessel wall is rigid, and the elasticity of the blood vessel is ignored. Previous studies have compared hemodynamic outcomes for rigid and elastic intracranial arteries, and there have been modest differences between the two hypotheses (Castro et al., 2015; Taylor et al., 1999; Albadawi et al., 2021). In the future, numerical simulation work will be carried out based on individualized models and boundary conditions to explore the impact of arterial stenosis and absence on cerebral hemodynamics and deepen our understanding of it.

In conclusion, this study contributes valuable insights into how vascular variations influence cerebral blood circulation regulation. These findings hold potential implications for optimizing treatment strategies and surgical planning for patients with cerebral ischemia, highlighting the importance of personalized approaches in clinical practice. Further research integrating more complex models and patient-specific data is warranted to deepen our understanding of CoW dynamics under varying pathological conditions.

## Data Availability

The original contributions presented in the study are included in the article/supplementary material, further inquiries can be directed to the corresponding author.
